# A novel application of bubble-eye strain of *Carassius auratus* for ex vivo fish immunological studies

**DOI:** 10.1038/s41598-021-89882-1

**Published:** 2021-05-24

**Authors:** Hiroto Nakajima, Atsushi Miyashita, Hiroshi Hamamoto, Kazuhisa Sekimizu

**Affiliations:** 1 Institute of Medical Mycology, Teikyo University, Tokyo, Japan; 2 Genome Pharmaceuticals Institute Co., Ltd, Tokyo, Japan; 3 Present Address: Drug Discoveries by Silkworm Models, Faculty of Pharma-Science, Teikyo University, Tokyo, Japan

**Keywords:** Applied microbiology, Pathogens

## Abstract

In this study, we investigated a new application of bubble-eye goldfish (commercially available strain with large bubble-shaped eye sacs) for immunological studies in fishes utilizing the technical advantage of examining immune cells in the eye sac fluid ex vivo without sacrificing animals. As known in many aquatic species, the common goldfish strain showed an increased infection sensitivity at elevated temperature, which we demonstrate may be due to an immune impairment using the bubble-eye goldfish model. Injection of heat-killed bacterial cells into the eye sac resulted in an inflammatory symptom (surface reddening) and increased gene expression of pro-inflammatory cytokines observed in vivo, and elevated rearing temperature suppressed the induction of pro-inflammatory gene expressions. We further conducted ex vivo experiments using the immune cells harvested from the eye sac and found that the induced expression of pro-inflammatory cytokines was suppressed when we increased the temperature of ex vivo culture, suggesting that the temperature response of the eye-sac immune cells is a cell autonomous function. These results indicate that the bubble-eye goldfish is a suitable model for ex vivo investigation of fish immune cells and that the temperature-induced infection susceptibility in the goldfish may be due to functional impairments of immune cells.

## Introduction

Infection control is key in aquaculture^1^. Failure to control infection can lead to colony collapse (i.e., death), which can be very damaging to the local industry. Despite significant investments in infection control, including the use of antibiotics, the aquaculture industry is still affected by infectious diseases^1–4^. Rapid temperature rises, often caused by heat waves, increase the risk of infection through a combination of accelerated pathogen growth and suppression of host immune defenses^1,5,6^. Understanding such complex host–pathogen interactions requires efficient models; models that allow in vivo, in vitro, and ex vivo studies will accelerate research in fish immunology and contribute to the fight against infectious diseases.

Temperature alteration impairs fish immunity and develops infections as evidenced by increased infection rates and shortened lifespan in aquatic species^5,7–13^. Most of those studies reported that a warm temperature is associated with increased infection resistance, while it is not always true for some contexts. Increased temperature triggers a stress response that suppresses the immune system of fish species^1^, and such hyperthermia may impact the fishery. To prevent potential damage to the industry, immunostimulants and vaccination may be effective, if not complete^14,15^. For such immunostimulant screening, it is essential to use both live animals raised in laboratory tanks and ex vivo analysis using freshly harvested immune cells. Ex vivo systems allow high-throughput screening of potent immunostimulant candidates, while in vivo confirmatory experiments are required for extensive testing at the industrial level. Lack of either approach will delay the research. In this study, we propose to use bubble eye goldfish to fulfill this requirement. Goldfish can be kept in a wide temperature range (typically 15–30 °C) ^16^, which is suitable for studying the effects of acute temperature changes on the immune system of goldfish.

The bubble-eye goldfish is a commercially available strain of goldfish in East Asia. Bubble-eye goldfish have large eye sacs (ocular sacs) under each eye. The eye-sac is surrounded by membranous epithelial cells with blood vessels running through the structure, and lymphatic fluid containing immunocompetent cells flows into the inner part of the sac. The sacs make it possible for experimenters to retrieve the immune cells using a needle without sacrificing the animal. The ocular sacs can be sampled repeatedly, allowing for time-course studies that track the same set of individuals over time. The lymph of the eye sac contains growth-promoting factors that act on fish cell cultures^17^, and cells in the eye sac fluid may play an immunological role. In this study, we will demonstrate the expression of immune-related genes in eye sac cells in response to immune challenge with heat-killed bacteria to elucidate their immune function. We will also demonstrate how temperature elevation affects immune function in goldfish, both in vivo and ex vivo, using a bubble eye goldfish model.

## Results

### Temperature rise promotes infection in the common goldfish

**Figure 1:**
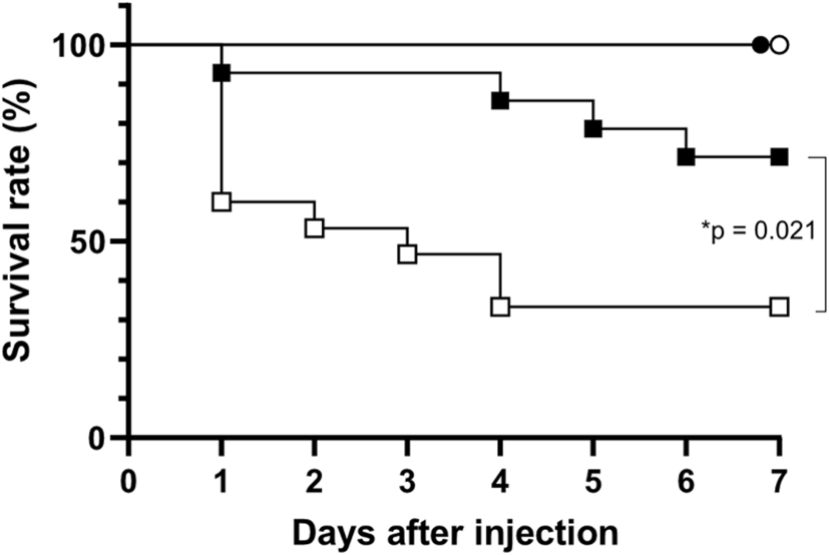
Temperature sensitivity of common goldfish for *Pseudomonas* infection*.* After the acclimation period, the common goldfish were divided into four groups as descried in the Materials and Methods section (two infection paradigms and two temperature paradigms). The goldfish were either infected (*P. aeruginosa*; 3 × 10^7^ CFU) or uninfected (saline) at either 25 $^\circ$C or 33 $^\circ$C. Survival curve for each group is shown in the figure over 7 days after the infection. Results from two experiments were combined and shown in the figure. Symbols: ● (saline, 25℃, n = 8), ○ (saline, 33℃, n = 9), ■ (*P. aeruginosa* infection, 25℃, n = 14) and □(*P. aeruginosa* infection, 33℃, n = 15). There was a statistically significand difference between the survival curves of *P. aeruginosa* infection (25ºC) and *P. aeruginosa* infection (33 $^\circ$C) groups (P = 0.021 by Log-rank test).

As shown in Fig. 1, the common goldfish injected intraperitoneally with *P. aeruginosa* died earlier when kept at elevated temperature (33 ℃) than when kept at the normal temperature (25 ℃). The two groups were statistically significantly different (p = 0.021, Log-rank test). No death was observed for the saline-injected groups (Fig. 1).

### Inflammatory responses to P. aeruginosa in the bubble-eye goldfish eye-sacs

**Figure 2:**
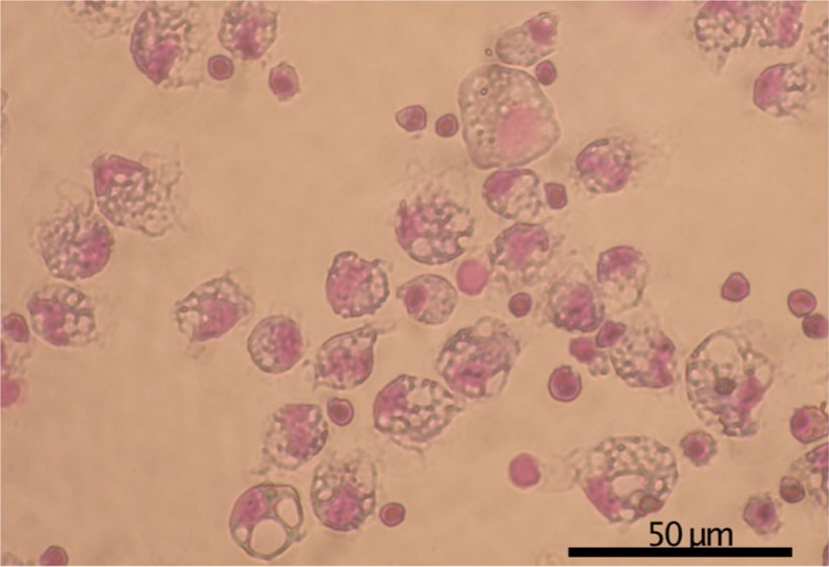
Microscopy image of the eye-sac cells from the bubble-eye goldfish. Eye-sac cells were harvested from eye sacs of untreated bubble-eye goldfish and stained with Giemsa. The scale bar represents 50 µm.

**Figure 3:**
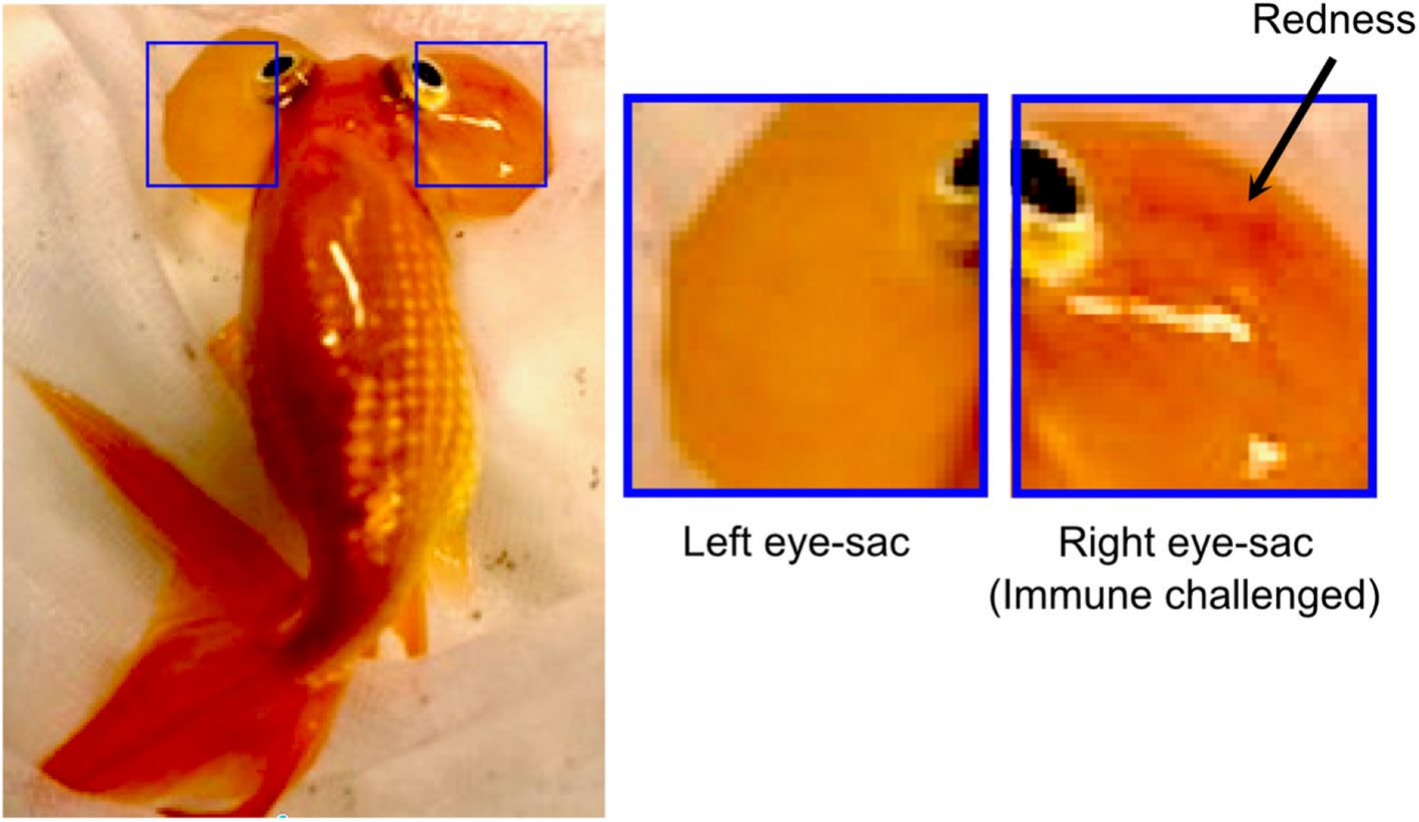
Inflammatory symptom (redness) of the eye sac. Heat-killed *P. aeruginosa* cells (50 µL of tenfold concentration of full growth) were injected into the right eye sac of the bubble-eye goldfish, while saline (50 µL) was injected into the left eye sac. Redness was observed on the right eye sac as shown in the figure after 20 h.

**Figure 4:**
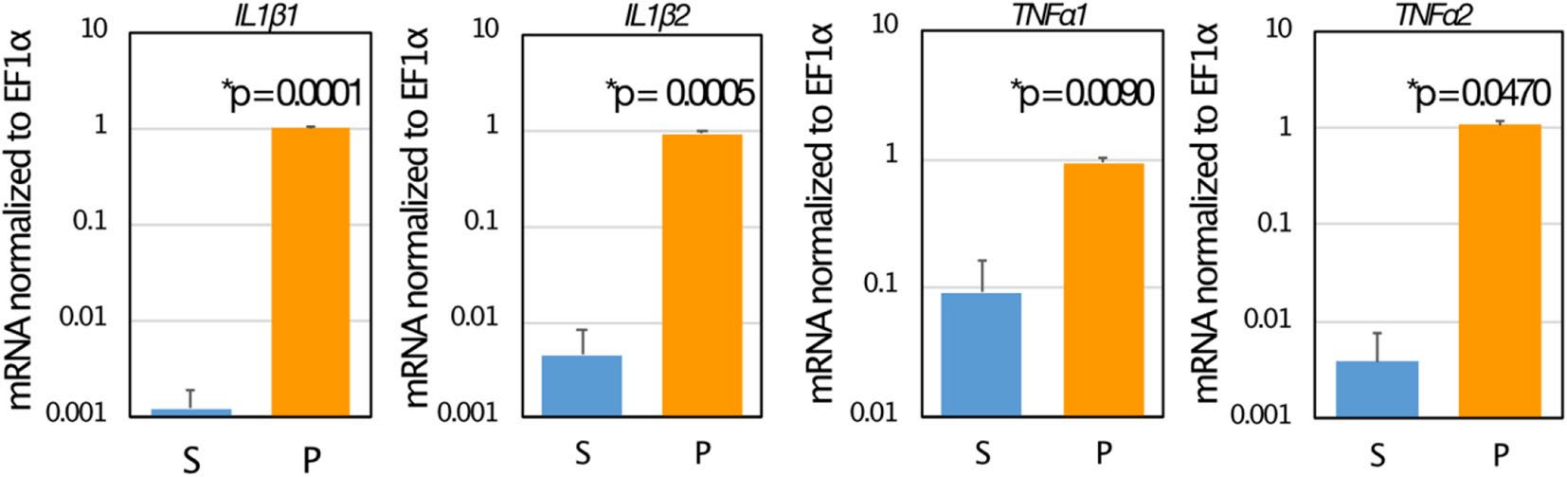
Pro-inflammatory cytokines are expressed in the eye-sac cells in the response to heat-killed *P. aeruginosa* cells. Twenty-four hours after the injection of heat-killed *P. aeruginosa* cells (50 µL of tenfold concentration of full growth; P) or saline (50 µL; S) into eye sac, eye sac cells were collected and analyzed for gene expression (see Materials and Methods section for details). Levels of mRNA for IL1β1, IL1β2, TNFα1 and TNFα2 were shown in the figure (values were normalized by a housekeeping gene (EF1α)). A representative result from three replicates is shown.

Eye sac cells (Fig. 2) can be harvested and cultured ex vivo. In the response to immune challenge by heat-killed *P. aeruginosa*, redness (prominent blood vessels) was observed in the ipsilateral eye sac membrane, but not in the contralateral membrane (Fig. 3), which represents an inflammatory response induced in the eye-sac. We further examined the gene expressions of pro-inflammatory cytokines in the eye-sac immune cells (see Materials and Methods for technical details). As shown in Fig. 4, the mRNA level increased by 800 fold for IL1β1 (p = 0.0001), 200 fold for IL1β2 (p = 0.0005), tenfold for TNFα1 (p = 0.0090), and 300 fold for TNFα2 (p = 0.0470) in response to the immune challenge (Fig. 4).

### Temperature rise suppresses the pro-inflammatory cytokine expressions in the eye-sac immune cells both in vivo and ex vivo

**Figure 5:**
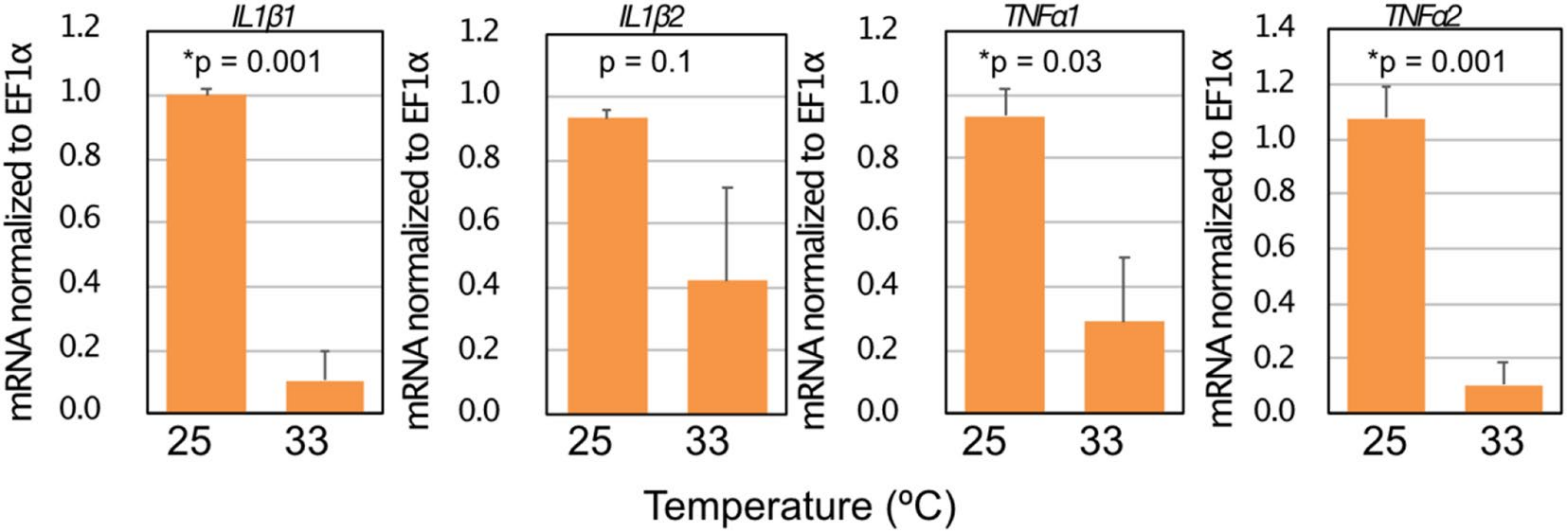
High temperature suppresses the expression level of pro-inflammatory cytokines in the eye-sac cells. Levels of mRNA for IL1β1, IL1β2, TNFα1 and TNFα2 were measured by qRT-PCR. Results from the increased-temperature (33 $^\circ$C, n = 3) condition and the normal temperature condition (25 $^\circ$C, n = 2) are shown in the figure (values were normalized to EF1α). P values from Student’s t-test are shown in the figure. The asterisks represent statistically significant differences between temperatures (significance levels were corrected by Benjamini–Hochberg procedure).

**Figure 6:**
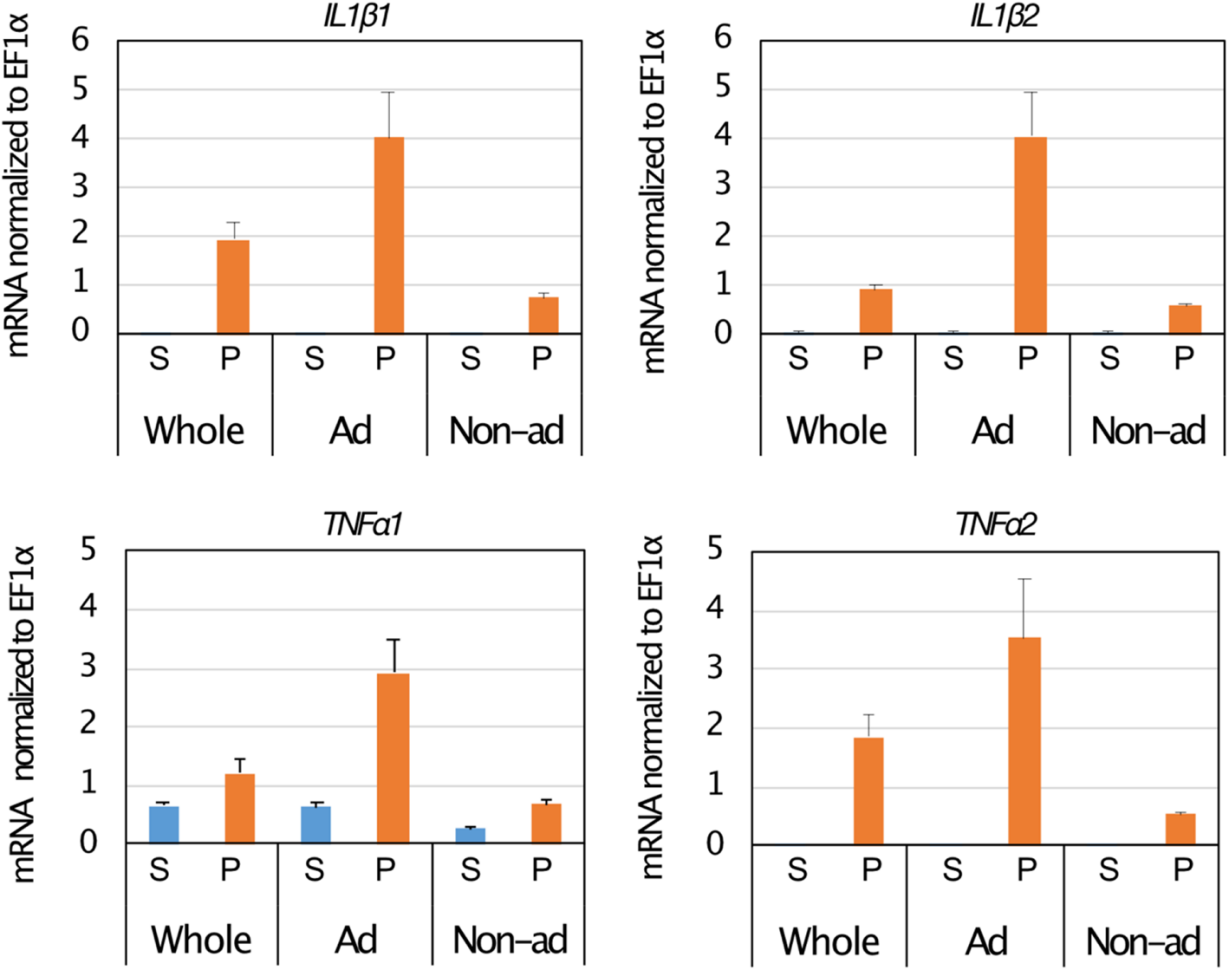
Ex vivo stimulation of eye-sac cells using heat-killed *P. aeruginosa* cells. The eye-sac cells of bubble-eye goldfish were suspended in medium (see Materials and Methods for details) and pre-incubated at 25 ℃ in 24-well plate (8 × 10^4^/0.8 mL/well) for two hours. Then, adherent (Ad) and non-adherent (Non-ad) cells, were separated by gentle pipetting and cultured. The result from unseparated cell population (Ad + Non-ad; Whole) are also shown in the panels. Gene expressions of pro-inflammatory cytokines in response to heat-killed *P. aeruginosa* (P) or saline (S) (four hours after adding of the *Pseudomonas* cells or saline into the culture media) are shown in the figure. The values were normalized to EF1α.

**Figure 7:**
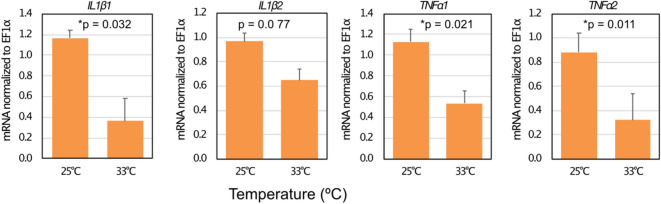
Temperature sensitivity of the ex vivo function of the eye-sac cells. Eye-sac cells, collected from three bubble-eye goldfish were cultured either at 25 $^\circ$C or 33 $^\circ$C. Gene expressions of pro-inflammatory cytokines in the presence of heat-killed *P. aeruginosa* cells (four hours after adding of the *Pseudomonas* cells) are shown in the figure. The values were normalized to EF1α. P values obtained from Student’s t-tests are shown in the figure. The asterisks represent statistically significant differences (significance levels were corrected by Benjamini–Hochberg procedure).

In the eye-sac immune cells in vivo, mRNA levels of induced pro-inflammatory cytokine genes were lower when the fish were kept at elevated temperature (33 ℃) than when kept at the normal rearing temperature (25 ℃) (Fig. 5). We then harvested the eye-sac immune cells from untreated bubble-eye goldfish to obtain an ex vivo culture at 25 ℃ (see Materials and Methods for details), where induction of pro-inflammatory cytokine genes by heat-killed *P. aeruginosa* was observed in adherent cells but not apparent in non-adherent cells (Fig. 6, and Fig. S4). Using the ex vivo culture, we examined the effect of temperature rise on the pro-inflammatory cytokine expressions in the eye-sac cells induced by heat-killed *P. aeruginosa* cells. Compared with the normal temperature, increased culture temperatures resulted in reduced cytokine expressions (Fig. 7, and Fig. S1–3).

## Discussion

In the present study, we established a model system to study immune responses using the bubble-eye goldfish. As demonstrated in the present study, this model is suitable for both in vivo and ex vivo analyses of immune cells. For the ex vivo experiments, the eye sacs allow us to collect immune cells without sacrificing the animal, and the harvested immune cells can be cultured on a plastic dish. The ex vivo cultured cells expressed cytokine genes in response to the bacterial challenge, which is consistent with the cytokine gene expressions triggered by an immune challenge observed in vivo. The immune cells showed attenuated expressions of cytokine genes in response to temperature rise as observed in vivo and ex vivo, which sheds light on the underpinning mechanism of the increased infection risks in aquaculture upon temperature rises.

Among the cytokines, IL1β and TNFα play important roles both in innate and acquired immunity, such as the activation of phagocytic cells and the promotion of immune-related gene expressions in a series of immunoreactive cells both in mammals and fishes^18^, indicating that whether an animal is capable of inducing the expression of IL1β or TNFα in response to immune challenges reflects its infection resistance. In this sense, the suppressed induction of these cytokines at elevated temperatures may explain the enhanced bacterial infection of goldfish in this experimental temperature increase, but the optimal temperature for bacterial growth may also be an important factor in assessing resistance.

The microscopy suggests that most of the eye-sac cells are heterogeneous in multiple histological properties such as cell size and nucleocytoplasmic ratio. It should be noted that the adhesion rate to the plastic dish was approximately 50%, and those cells that did not adhere to the dish may represent distinct population from the adherent cell populations. At least, the adherent population showed apparent expressions of pro-inflammatory cytokines in response to bacterial challenge, suggesting its role in the pro-inflammatory cytokine production. Also, mammalian studies revealed that dish-adhering cell populations are rich in monocyte/macrophage lineage cells^19,20^, while goldfish macrophages express IL1β and TNFα^21^. These are consistent with our finding, and the dish-adhering eye-sac immune cells are most likely monocyte/macrophage lineage cells that respond to the immune challenge by expressing the pro-inflammatory cytokines.

Goldfish are eurythermic^16^ and often kept in a wide range of temperatures (usually 15 to 30 ℃). Goldfish can tolerate temperature rise after enough period of acclimation^22^. In our experiments, physiological conditions of goldfish were not investigated but no apparent changes were made between at normal (25 ℃) and at increased temperature (33 ℃). Regarding the temperature sensitivity of cytokine expressions, empirical knowledge in fish immune cells is slim. A very recent in vivo study in the crucian carp demonstrated that the host viability and the gene expression of pro-inflammatory cytokines after a bacterial infection was reduced by increased temperature^23^, supporting the present study in the bubble-eye goldfish. In the bubble-eye goldfish, the eye sac enables immune cell analyses as it provides up to 1 mL of lymphoid fluid from each individual, even without sacrificing the animal. This feature is the major advantage of this model using the bubble-eye goldfish and will accelerate the molecular study of fish immunity and contribute to improved aquaculture productivities.

## Methods

### Goldfish strains (Carassius auratus)

The common goldfish (‘Wakin’) and the bubble-eye goldfish were obtained from a local supplier Kingyo-Zaka (Tokyo, Japan). Goldfish were fed with a commercial diet for goldfish (Kyorin, Hyougo, Japan). All experiments were done after an acclimation period (more than a week) in the laboratory after each purchase, where we kept the goldfish at 25 ℃ in a fish tank (30 × 45 × 23 cm). The ranges of body weight were 7–10 g for the common goldfish, and 20–28 g for the bubble-eye goldfish upon the start day of each experiment. The research protocol was approved by Animal Welfare Ethics Committee of Genome Pharmaceuticals Institute Co., Ltd. and was conducted in compliance with all relevant guidelines and regulations applicable at the time and place of the experiments, including the ARRIVE guidelines.

### Temperature rise paradigms

Temperature rise paradigms were given to the goldfish following the acclimation period at the normal temperature (25 ℃). For the infection experiments and the sterile immune challenge experiments, goldfish were kept at either 25 ℃ or 33 ℃ for 24 h before treatments (i.e., infection or sterile challenge). For ex vivo experiments using harvested eye-sac immune cells, cells were harvested from bubble-eye goldfish reared at 25 ℃, and the harvested cells were cultured either at 25 ℃ and 33 ℃.

### Bacteria (Pseudomonas aeruginosa)

*P. aeruginosa*, strain PAO1^24^ was aerobically cultured overnight at 37 ℃ in LB10 medium. In infection experiments, the live *P. aeruginosa* cells were washed and suspended in saline (0.9% NaCl aqueous solution). For the heat-killed *P. aeruginosa* cells used in this study, we washed the live cells with saline, and then autoclaved the cells at 121 ℃ for 20 min.

### Infection experiments

To know the effect of temperature on the goldfish immunity, we intraperitoneally injected *P. aeruginosa* live cells (3 × 10^7^ CFU/fish) to the common goldfish (Wakin). The injected fish were then kept at either 25 ℃ (normal rearing temperature) or 33 ℃ (increased temperature) and monitored for their survival.

### Sterile immune challenge to the eye-sac using heat-killed bacterial cells

An overnight culture of *P. aeruginosa* was spun and the pellet was resuspended in a tenth volume of saline. We autoclaved this suspension at 121 ℃ for 20 min to obtain heat-killed *P. aeruginosa* cells. We injected 50 µL of the heat-killed *P.* aeruginosa cells into the eye-sac of one side, and 50 µL of saline into the eye-sac of the other side. The immune challenges were done either at the normal rearing temperature (25 ℃) or at the increased temperature (33 ℃).

### Collection of eye-sac cells

Because the common goldfish is difficult to collect their immune cells without sacrificing the animal, we used the bubble-eye goldfish in the following part of this study to investigate the molecular responses of the goldfish immune system to temperature rises. Eye-sac cells floating in the eye-sac fluid can be easily collected from the eye sacs of bubble-eye goldfish (≧2 × 10^5^ cells/mL). Eye-sac cells consisted of cells of various sizes, ranging from 5 to 20 µm, and different nucleocytoplasmic ratios (Fig. 2). Some of the cells showed adhesion to plastic dishes in vitro (Fig. S4). From the bubble eye-goldfish, the eye-sac fluid (containing the eye-sac cells) was collected from the eye sacs using a disposable plastic syringe with a 21-Gauge sterile needle.

### Gene expression analysis of eye-sac immune cells

Twenty-four hours after the injection of heat-killed *P. aeruginosa* into the eye sac, we collected the eye-sac cells as described in the ‘Collection of eye-sac cells’ section. We then isolated the mRNA and performed qRT-PCR analyses (details describe in the following section).

### Ex vivo culture of the eye-sac cells

We used a disposable syringe (5 mL) with needle (21 Gauge) to harvest the eye-sac immune cells. The harvested cells (typically 4 mL) were suspended in RPMI 1640 medium (Sigma-Aldrich) supplemented with 10% heat-inactivated calf serum (SAFC Biosciences, USA), 3% autologous eye-sac fluid, 20 mM HEPES buffer and antibiotics (100 U/mL of penicillin and 100 µg/mL of streptomycin) and cultured in 24-well plastic plate (catalog# 3820–024, AGC Techno Glass) (8 × 10^4^ cells/0.8 mL/well) for at least 2 h. The medium had been preincubated at 25 ºC. In this condition, approximately 50% of the harvested cells adhered to the plastic dish. To give an immune challenge, 1.5 × 10^8^ cells of heat-killed *P. aeruginosa* were added in the medium when the cells were suspended in the culture dish.

### RNA isolation and quantitative real-time polymerase chain reaction (qRT-PCR)

Total RNA was isolated from eye-sac cells by using TRIzol Reagent (ThermoFisher Scientific), treated with DNase I (Promega), and reverse transcribed to obtain cDNA using the High Capacity RNA-to-cDNA Kit (ThermoFisher Scientific) following the manufacturer’s protocol. Using the cDNA, gene expressions were analyzed by qRT-PCR. The qRT-PCR was performed (relative standard method using a standard curve for each target), using 7500 Fast Real-Time PCR System (ThermoFisher Scientific) and Fast SYBR Green Master Mix (ThermoFisher Scientific). Primers for each target gene were described in the literature^21^. We chose the elongation factor 1α (EF1α) gene as an internal control as done in the preceding study in the goldfish^21^. As reported in the literature^25^, the goldfish has L-type and S-type ohnologs. The target genes we analyzed in this study were either on S-type or L-type chromosome. Further information is summarized in Supplementary Table S1.

### Statistical analysis

To test the differences between survival curves, log-rank tests were performed using GraphPad Prism version 8.4.3 (GraphPad Software Inc.). To test the differences between mean values, Student’s t-tests were performed using Microsoft Excel 2013.

## Supplementary Information

Supplementary Information.
