# Supplementary material for: A novel application of bubble-eye strain of Carassius auratus for ex vivo fish immunological studies
Source: Sci Rep. 2021 May 24;11:10757. doi: 10.1038/s41598-021-89882-1 (PMC8144383; doi:10.1038/s41598-021-89882-1)
Supplement: Supplementary file 1 — Supplementary Information. [file 41598_2021_89882_MOESM1_ESM.docx]

**Supplementary Information for:**

**A novel application of bubble-eye strain of *Carassius auratus* for *ex vivo* fish immunological studies**

**Hiroto Nakajima^1,2,3^, Atsushi Miyashita^1^, Hiroshi Hamamoto^1^, and Kazuhisa Sekimizu^1,2,3,*^.**

1. Institute of Medical Mycology, Teikyo University, Tokyo, Japan.

2. Genome Pharmaceuticals Institute Co., Ltd., Tokyo, Japan.

3. Drug Discoveries by Silkworm Models, Faculty of Pharma-Science, Teikyo University, Tokyo, Japan (present affiliation).

***** sekimizu@main.teikyo-u.ac.jp

**
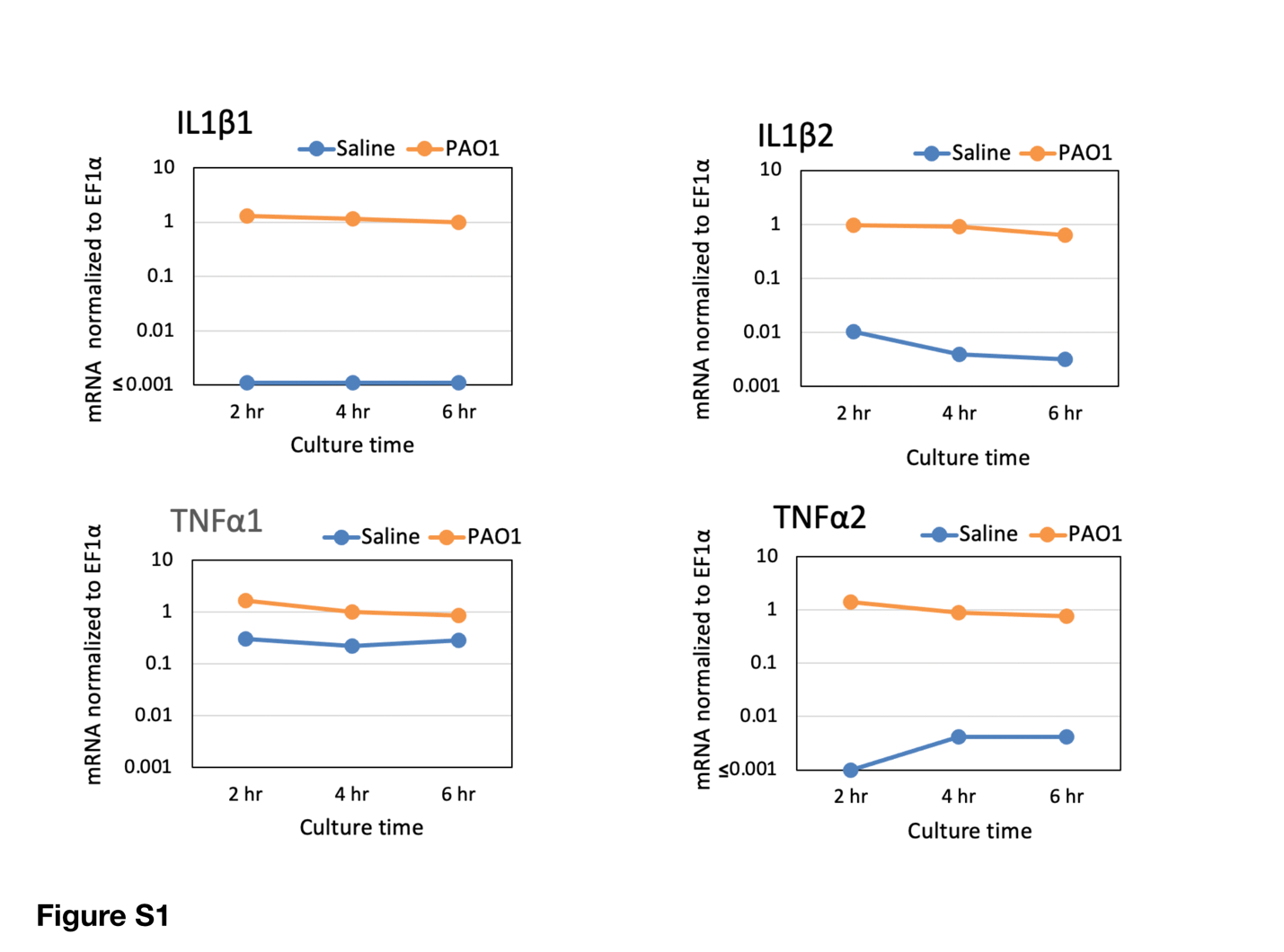
**

**Figure S1. Stable expression of pro-inflammatory cytokines in *ex vivo* eye-sac cell culture.** Eye-sac cells collected from bubble-eye goldfish were cultured with heat-killed *P. aeruginosa* (PAO1) or saline at 25℃ (8 x 10^4^/0.8 mL/well). Gene expressions of pro-inflammatory cytokines at 2, 4, and 6 hours after the adding of heat-killed *P. aeruginosa* cells or saline into the media are shown in the figure. The values were normalized to EF1α. Shown is the representative result of multiple (typically three) experimental replicates.


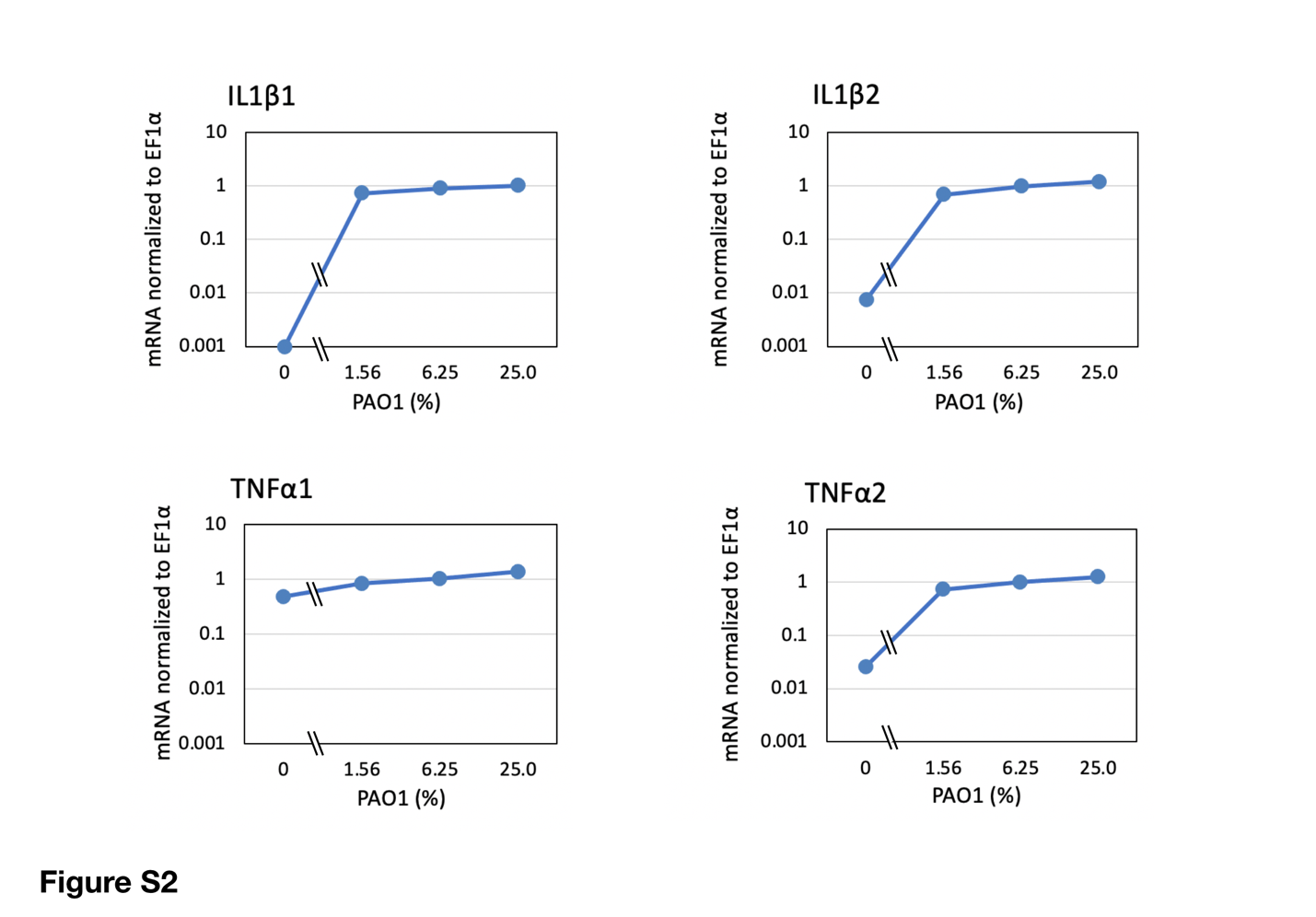


**Figure S2. Dose-dependent *ex vivo* induction of pro-inflammatory cytokines in the eye-sac cells.** Eye-sac cells collected from bubble-eye goldfish were cultured with heat-killed *P. aeruginosa* (PAO1; three doses in the figure) or saline (0% in the figure) at 25℃ (8 x 10^4^/0.8 mL/well). Gene expressions of pro-inflammatory cytokines are shown in the figure. The values were normalized to EF1α. Shown is the representative result of multiple (typically three) experimental replicates.

**
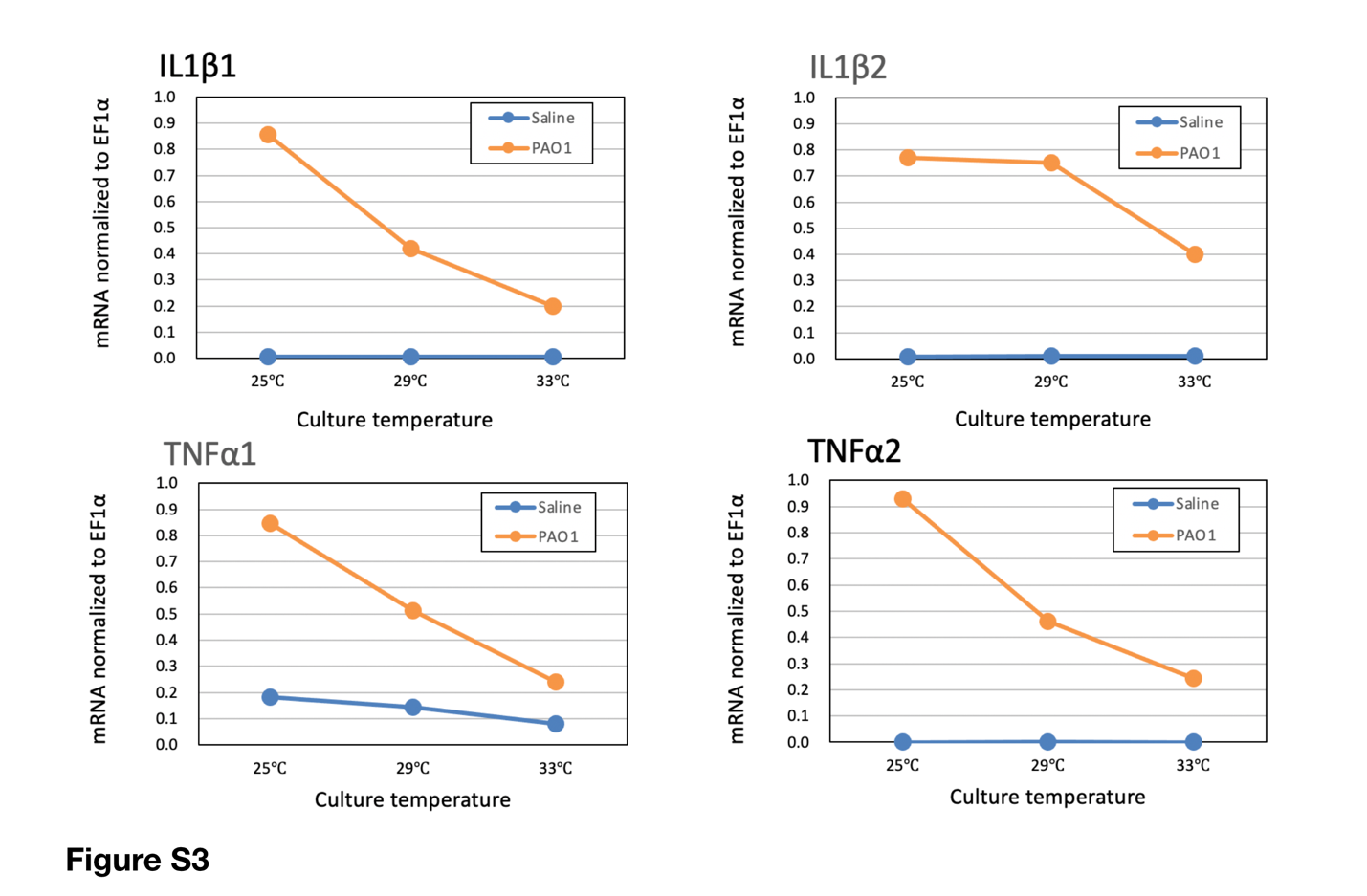
**

**Figure S3. Temperature-dependent suppression of pro-inflammatory cytokine induction of the eye-sac cells.** Eye-sac cells collected from untreated bubble-eye goldfish were cultured with heat-killed *P. aeruginosa* or saline at 25ºC, 29ºC, or 33ºC as indicated in the figure for four hours (8 x 10^4^/0.8 mL/well). Gene expressions of pro-inflammatory cytokines are shown in the figure. The values were normalized to EF1α. Shown is the representative result of multiple (typically three) experimental replicates.


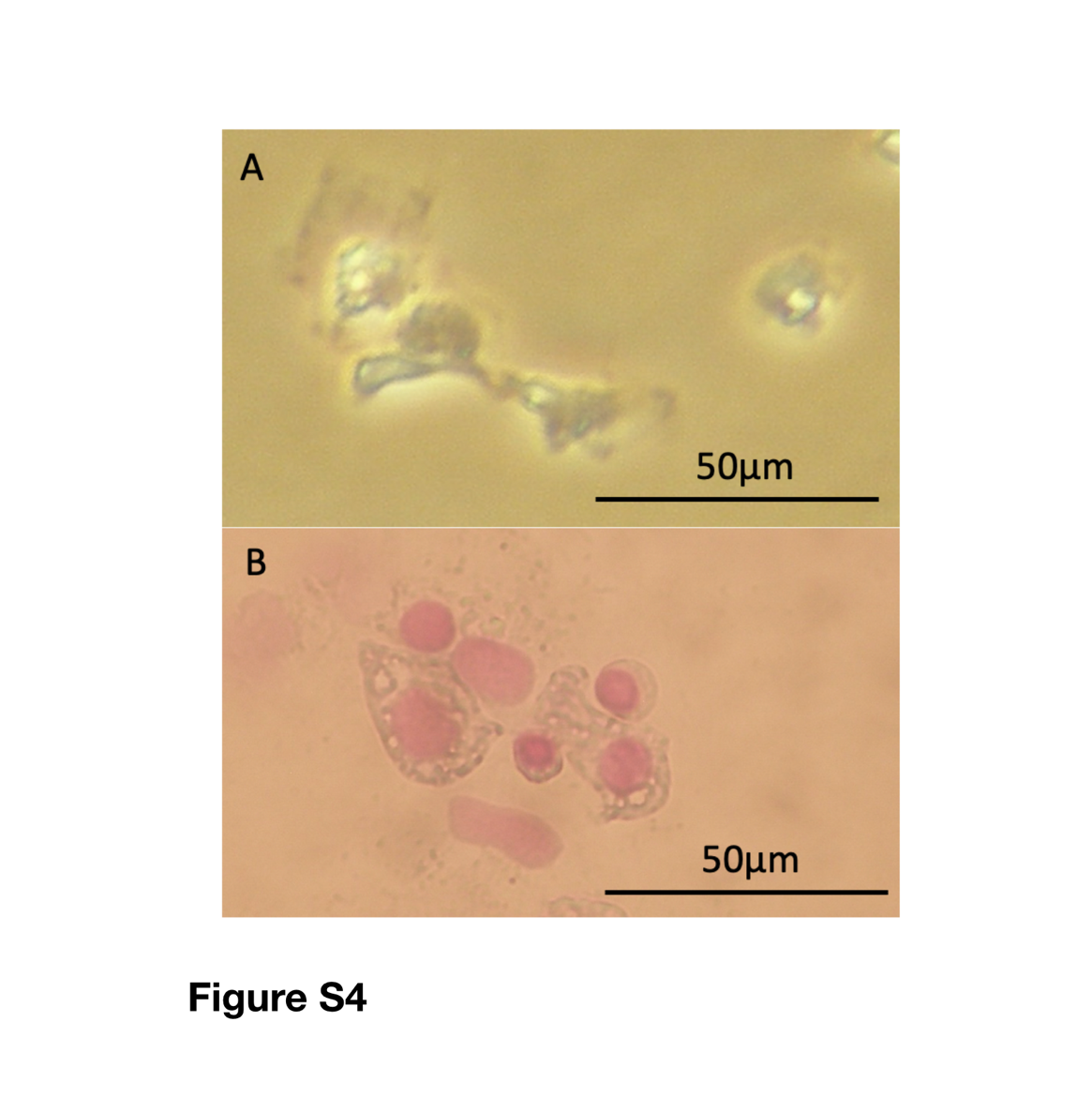


**Figure S4. Microscopy image of the adherent eye-sac cells from the bubble-eye goldfish.** Eye-sac cells were harvested from eye sacs of untreated bubble-eye goldfish and cultured at 25 ºC for 2 hours in 24-well plastic plate (A) or 8-well microscope glass slide (B). Non-adherent cells were removed by gentle pipetting. Adherent cells were photographed microscopically either by an inverted microscope (A), or after Giemsa staining (B). The scale bars represent 50µm.

| Target | Gene Locus | S- or L- chromosome |
| --- | --- | --- |
| EF1alpha | Chromosome 19 | L |
| IL1beta-1 | Chromosome 10 | L |
| IL1beta-2 | Chromosome 35 | S |
| TNFalpha-1 | Not found | - |
| TNFalpha-2 | Chromosome 19 | L |

Table S1. The target genes analyzed in this study.

The five target genes are either on the L- or S- chromosomes as reported in Kon et al. 2020 ^1^.

1 Kon, T. *et al.* The Genetic Basis of Morphological Diversity in Domesticated Goldfish. *Curr Biol* **30**, 2260-2274 e2266, doi:10.1016/j.cub.2020.04.034 (2020).
